# Elucidating β‑Sheet Ordering in Lipopeptides Bearing Lysine-Rich Tripeptide Sequences: Fibrils versus Nanotapes

**DOI:** 10.1021/acs.jpcb.5c06441

**Published:** 2025-12-18

**Authors:** Ian W. Hamley, Valeria Castelletto, Mario Tagliazucchi

**Affiliations:** † School of Chemistry, Food Biosciences and Pharmacy, 6816 University of Reading, Whiteknights, Reading RG6 6AD, U.K.; ‡ INQUIMAE-CONICET and DQIAQF, University of Buenos Aires, School of Sciences, 62873 Ciudad Universitaria, Pabellón 2, Ciudad Autónoma de Buenos Aires, Buenos Aires C1428EHA, Argentina

## Abstract

The self-assembly in aqueous solution and conformation of lipopeptides C_16_-WKK, C_16_-KWK, C_16_-YKK and C_16_-KYK is compared and examined. Remarkable differences are observed among the systems despite the small sequence changes comparing C_16_-XKK with the C_16_-KXK homologue (X = W or Y), depending on pH. These are rationalized using a molecular theory for amphiphile self-assembly (MOLT) to predict the morphology along with atomistic molecular dynamics simulations to probe local conformation and packing, along with new experimental data from small-angle X-ray scattering (SAXS) and FTIR spectroscopy. MOLT correctly describes the high-pH morphology behavior, i.e., fibrils for C_16_-XKK, and lamellar nanotapes for C_16_-KXK, although it predicts micelles for all systems at low pH, whereas experiments indicate that this only occurs for the C_16_-XKK lipopeptides, not the C_16_-KXK, which form lamellar nanotapes stable over an extended range of pH 2–12. Atomistic MD reveals β-sheet conformation is more favored for the C_16_-XKK lipopeptides which also have enhanced aggregation propensity compared to C_16_-KXK analogues. The extent of π-stacking was higher for the latter lamellar nanotape structures. The extent of hydrogen bonding is higher for the tyrosine-containing molecules than the tryptophan-based ones. The combination of a molecular theory and atomistic MD provides a comprehensive insight into the remarkable sequence- and pH-dependent molecular ordering within these model lipopeptides which will enable the rational design of future peptide amphiphiles with targeted nanostructures for desired applications.

## Graphical abstract

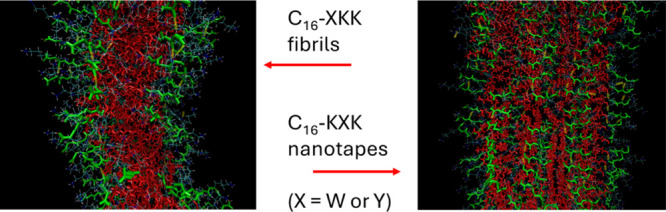

## Introduction

Understanding β-sheet fibril formation of peptides and lipopeptides is a significant challenge motivated by the extensive range of applications of such structures as structural or functional biomaterials,^1–9^ and their potential role in the treatment of conditions including amyloid diseases^10–13^ and others.^14–18^ As well as studies using natural or nature-derived sequences, much attention has been dedicated to develop model peptides and lipopeptides to study β-sheet formation through experimental, simulation, machine learning/AI and theoretical methods.

There has been considerable recent progress in the successful use of molecular dynamics (MD) to model lipopeptide and peptide β-sheet fibril formation. In an early example of a study on fibril-forming lipopeptides, Schatz and co-workers showed that atomistic MD can successfully be used to model the formation of cylindrical fibrils by the lipopeptide C_16_-SLS­LAA­AEI­KVAV.^19^ The simulations were based on fibrils constructed as radially arranged lipopeptides in disks (of 9 molecules) with an angular displacement of the chains along the fibril axis 16 times to give a total of 144 molecules. The simulations provide quantitative information on the fractions of different secondary structures present as well as other structural properties and information on residue-specific hydrogen bonding.^19^ In a companion paper, the properties of fibrils of C_16_-V_2_A_4_E_3_ and C_16_-V_4_A_2_E_3_ were compared by atomistic MD, and while both form similar cylindrical fibrils, the extent of β-sheet formation is higher for the valine-rich lipopeptide.^20^ This correlates to observed experimental properties,^21^ and this work was further developed to model the self-assembly of a lipopeptide into fibrils using coarse-grained MD (CG-MD).^22^ The same lipopeptide was modeled by mapping atomistic parameters onto those in the MARTINI coarse-grained force field. Fibril formation was observed over a time scale extending to 16 ms in the coarse-grained simulations using tens of molecules with coarse-grained parameters chosen to represent a mixture of random coil, β-sheet and α-helix conformations, as in earlier atomistic simulations.^22^

In another example, atomistic MD was used to model the formation of twisted β-sheets by the yeast Sup35 amyloid peptide GNN­QQNY.^23^ Starting from an initial configuration of 20 peptide pairs replicated along a fibril axis, the system evolved with fibril twist, the extent of which depends on the terminal charges of the peptide. Yarovsky and co-workers have performed atomistic MD simulations of lipopeptides containing β_3_-homoAla and lysine or arginine.^24^ The simulations were able to reproduce nanobelt and twisted fibril structures observed experimentally. The methodology used was first to create seeds for fibril formation using well-tempered metadynamics to enhance conformational sampling of 8 randomly orientated monomers. Following this, selected dimers or trimers from the seeds were used to create stacked assemblies (favoring stacked β-sheets along the fibril axis) by replication and rotation/stacking.^24^ Multiscale simulations have been performed to examine the fibril formation of amyloid peptides (7-mers to 11-mers based on natural amyloid sequences).^25^ The authors note that the secondary structure is constrained at the outset in coarse-grained simulations and that atomistic simulations are thus necessary to properly represent secondary structure development,^25^ which in the case of β-sheets implies intermolecular hydrogen bonding. They therefore complemented coarse-grained simulations of gross fibril morphology, with atomistic simulations to provide detail on local structure. Considering properties associated with fibril formation from experiments (such as β-sheet content from FTIR spectroscopy) and simulation quantities, they identified peptide aggregation propensity, i.e., the ratio of initial-to-final solvent-accessible surface area (SASA),^26^ as a key property relevant to aggregation that can be extracted from the simulations.^25^

The packing of the model amyloid peptide AAK­LVFF (containing a core fragment Aβ16-20 KLVFF from the amyloid β peptide) into parallel and antiparallel β-sheets with a range of modeled steric zipper structures with in- and out- of phase stacking was examined by atomistic MD.^27^ Antiparallel structures were favored, including one with linear twisted sheets. The modeled assemblies were used to compute circular dichroism spectra for comparison to experimental data. Monomer and dimer structures were also modeled by simulated annealing, using constraints from NMR NOE measurements.^27^ Atomistic MD has recently been employed to examine the formation of pleated and rippled β-sheets by short model linear and cyclic peptides and peptide enantiomer pairs, and the number of hydrogen bonds and the cohesive energy density were identified as key parameters.^28^

Atomistic MD simulations in explicit water were performed for lipopeptides C_12_-K and C_16_-K, starting from bilayers of randomly packed molecules.^29^ The focus of the simulations was to understand the effect of degree of ionization (experimentally controlled via pH) on the arrangement of the molecules in the bilayer, in comparison to WAXS data. The MD results confirmed that the molecules are tilted in the bilayers, the tilt angle and area per lipid depending on the degree of ionization.^29^ In a study on energy landscapes of lipopeptide fibrils, atomistic MD was performed for C_16_-V_3_A_3_K_3_.^30^ The simulations were based on the same model for related sequences, discussed above, for fibrils constructed as radially arranged lipopeptides in disks with an angular displacement of the chains along the fibril axis. With the aim to correlated molecular parameters from MD simulations to experimentally measured properties related to fibril shape and stability, Stupps’s group recently used course grained MD to sample 10,000 palmitoyl (C_16_-) lipopeptides with randomly generated sequences of 4–10 peptide residues.^31^ The lipopeptides were screened for fibril formation and those that form fibers were then selected for atomistic MD simulations based on small clusters of 25 molecules. Among the descriptors considered, the property that correlated best to experimental results was the number of hydrogen bonds per lipopeptide.^31^

The time scales involved in micellization (approximately s to ms)^32^ are still demanding for MD simulations, which makes it difficult to assess whether predicted structures are truly equilibrium states. As an alternative to MD, a mean-field molecular theory for amphiphile self-assembly, MOLT, was proposed to study the thermodynamics of lipopeptide aggregation.^33,34^ MOLT takes as inputs the molecular structure of the amphiphile (at a similar level of coarse-graining as CG-MD) and solution conditions (pH, ionic strength), and produces as an output the free energy and internal structure of aggregates of different shape (planar lamellar, cylindrical fibrils and spherical micelles) and aggregation number/density. The aggregate with the lowest free energy is predicted to be the most stable, equilibrium, structure. Moreover, acid–base chemical equilibria are explicitly accounted for within this formalism, allowing constant-pH predictions, which are difficult to obtain by MD simulations. Previous work has shown that MOLT can correctly predict the pH-dependent behavior of C_16_-KK, C_16_-KKK, C_16_-EE and C_16_-EEE, as well as mixtures of these lipopeptides.^33,35,36^ While MOLT provides a fast and computationally inexpensive prediction of the equilibrium morphology at a given pH, it lacks the level of detail provided by atomistic MD and (like CG-MD) its predictions strongly depend on the proper parametrization of the interactions. This work combines for the first time atomistic MD and MOLT to address the complex self-assembly behavior of lipopeptides.

Here, we also introduce a method to perform atomistic simulations of lipopeptide nanotape β-sheet assemblies that successfully reproduces the local fibril structure from SAXS and that can be used to identify key factors in the aggregation process. The method is able to discriminate between the self-assembly of homologous model cationic lipopeptide pairs C_16_-WKK/C_16_-KWK and C_16_-YKK/C_16_-KYK. In addition, the pH-dependent self-assembly is modeled using MOLT.

We compare the self-assembly of two pairs of model cationic lipopeptides, C_16_-KWK and C_16_-WKK and C_16_-KYK and C_16_-YKK (C_16_: hexadecyl or palmitoyl, K: lysine, W: tryptophan, Y: tyrosine). We have recently found that whereas C_16_-WKK and C_16_-YKK show marked antimicrobial activity against both Gram- negative and positive bacteria,^37,38^ the corresponding homologues C_16_-KWK and C_16_-KYK (with only a switch in the two C-terminal residues) show minimal antimicrobial activity. It is also notable from our prior reports that C_16_-KWK and C_16_-KYK show pH-dependent self-assembly, forming micelles at low pH 3, but extended β-sheet fibrillar or nanotape structures at higher pH 8.^38,39^ In contrast, C_16_-WKK and C_16_-YKK form stable β-sheet structures across this pH range. We therefore carried out detailed atomistic MD simulations to elucidate possible conformational and structural differences among these lipopeptides

## Methods

### Materials and Sample Preparation

Lipopeptides were purchased from BioservUK (Rotherham, UK) and supplied as TFA salts. The molar masses measured by ESI-MS are as follows: C_16_-KWK 699.55 g mol^–1^ (698.51 g mol^–1^ expected) and C_16_-KYK 676.50 g mol^–1^ (675.49 g mol^–1^ expected). The purity by HPLC (0.1% TFA in acetonitrile/water gradient) is 96.5% for C_16_-KWK and 95.5% for C_16_-KYK. Data for C_16_-WKK and C_16_-YKK were as reported previously.^38,39^ Solutions were prepared by dissolution in ultrapure water, samples pH 8 were prepared by addition of suitable amounts of 2 M NaOH solution.

### Small-Angle X-ray Scattering (SAXS)

SAXS experiments were performed on beamline B21 ^40^ at Diamond (Didcot, UK). The sample solutions were loaded into the 96-well plate of an EMBL BioSAXS robot and then injected via an automated sample exchanger into a quartz capillary (1.8 mm internal diameter) in the X-ray beam. The quartz capillary was enclosed in a vacuum chamber, to avoid parasitic scattering. After the sample was injected into the capillary and reached the X-ray beam, the flow was stopped during the SAXS data acquisition. Beamline B21 operates with a fixed camera length (3.9 m) and fixed energy (12.4 keV). The images were captured using a PILATUS 2 M detector. Data processing was performed using dedicated beamline software ScÅtter.

### FTIR

FTIR experiments were performed in solutions at pH 8, by dissolving the peptides in controlled amounts of D_2_O and 1 wt % NaOD. Because pH measure is not rigorous for samples containing D_2_O, FTIR samples were prepared using similar amounts of solid (peptide) and liquid (D_2_O and 1 wt % NaOD) components as those used to prepare the corresponding sample at pH 8 in water. Following preparation, a pH indicator stick was used to qualitatively check the pH 8 of the samples, and samples were left to rest for 24 h at 5 °C. FTIR spectra were obtained using a Thermo-Scientific Nicolet iS5 instrument with a DTGS detector. A 100 μL of solution was placed in a Specac Pearl liquid cell with CaF_2_ plates. Each FTIR spectrum was corrected by its corresponding D_2_O/NaOD spectra. For each sample, a total of 128 scans were recorded over the range of 900–4000 cm^–1^. Data for C_16_-YKK and C_16_-WKK are those reported previously.^38^

### Molecular Dynamics Simulations

Molecular dynamics simulations were performed using Gromacs^41^ (versions 2024.4, 2023.2 or 2020.1-Ubuntu-2020.1-1). Simulations were performed using the CHARMM27 force field^42,43^ using the included force field parameters for C_16_ (palmitoyl) chains which were manually adapted to build the lipid-peptide linking unit. Lys residues have charge +1 and Tyr and Trp are neutral, while the C terminus was represented as COOH. Hydrogen-bonded β-sheets were manually constructed by manually creating a dimer of lipopeptides with backbone hydrogen bonds and then successive doubling to a 4-mer, 8-mer, 16-mer and 32-mer, maintaining the parallel β-sheet hydrogen bonding pattern. For C_16_-KWK and C_16_-KYK, the 32-mers were then arranged to form palmitoyl bilayers in 64-mers with an interdigitated packing (Figure 1), which were then stacked by successive doubling (taking care to avoid clashes of terminal peptide groups) to give 256-mers (Figure 1) used as starting configurations. Each system was put into the principal axis in a box of size 19.86 nm × 7.95 nm × 5.96 nm, and the system was solvated using spc216 water. After energy minimization and 100 ps relaxation stages in the NVT ensemble, the final simulations were carried out in the NPT ensemble using a leapfrog integrator with steps of 1 fs up to 1 or 4 ns depending on the equilibration of the system. The temperature was maintained at 303.13 K using the velocity-rescale (modified Berendsen) thermostat^44^ with a coupling constant of 10 steps. The pressure was maintained at 1 bar using the Parinello–Rahman barostat^45^ and periodic boundary conditions were applied in all three dimensions. The Particle Mesh Ewald scheme^46,47^ was used for long-range electrostatics. Bonds were constrained using the LINCS algorithm^48^ and the Verlet cutoff scheme^49^ was used. Coulomb and van der Waals cutoffs were 1.0 nm.

**Figure 1:**
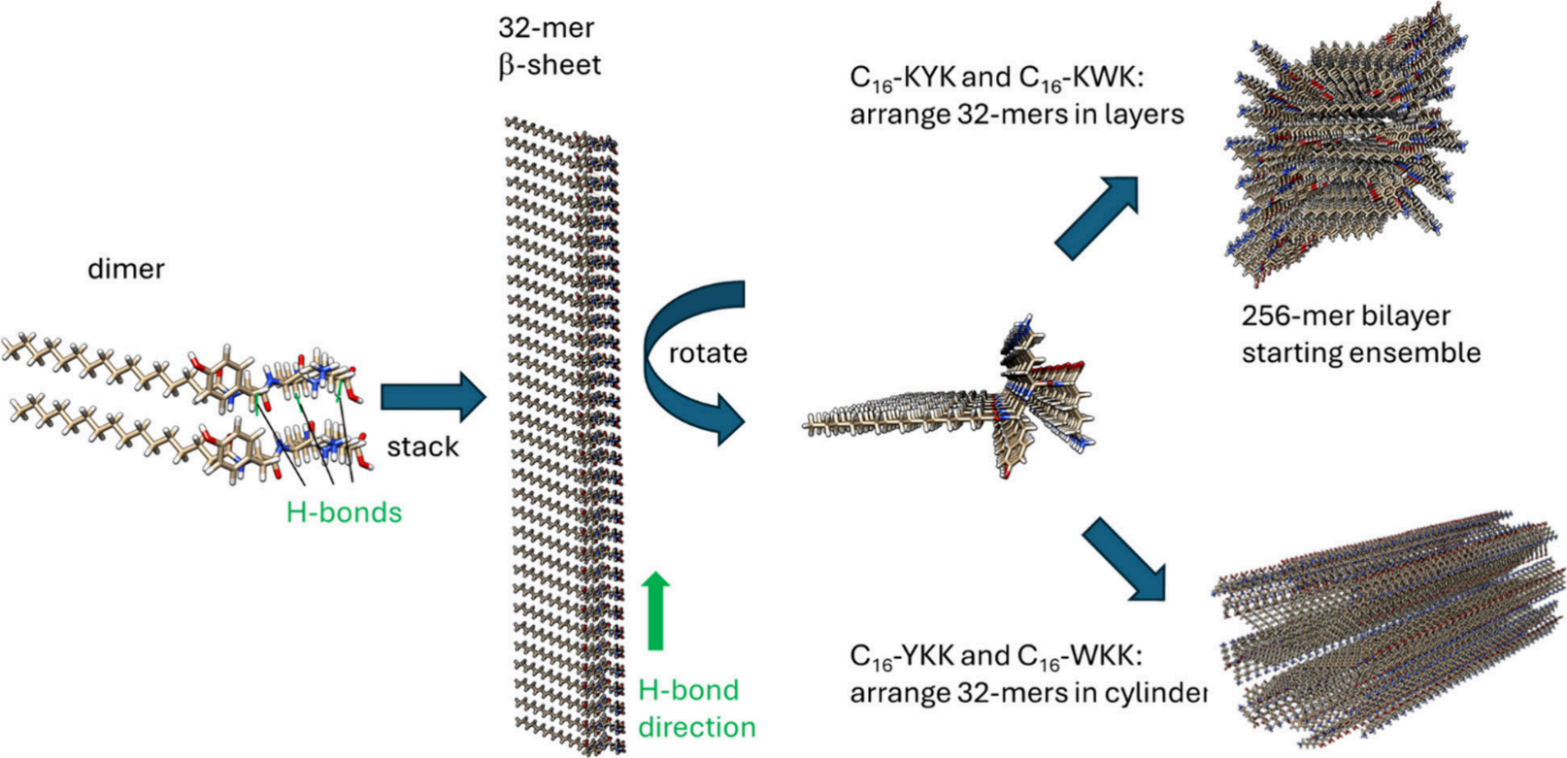
Method to generate starting configurations of β-sheet bilayers for MD simulations. A dimer with multiple H-bonds is created and then duplicated by translation along the H-bond direction to produce a 32-mer β-sheet. After rotating the structure to consider the lipid chains, for C_16_-KYK and C_16_-KWK they are arranged to produce a 256-mer β-sheet bilayer, whereas in C_16_-YKK and C_16_-WKK they are arranged along the axis of a cylinder. In both cases steric clashes are avoided while maintaining sufficiently close lipid packing.

### MOLT

The MOLT formalism has been described in detail previously,^33,36^ and we provide here only a brief description of the general concept of MOLT. The theory is formulated from a free-energy functional of the system, which contains contributions from the translational entropy of the lipopeptides, salt ions and water molecules; the conformational entropy of the lipopeptides; short-range attractions; electrostatic interactions and acid–base chemical equilibria. Intramolecular repulsions are explicitly considered, while intermolecular repulsions are incorporated with a mean-field packing constraint. Unlike most analytical theories for amphiphile self-assembly,^50^ MOLT explicitly takes into account the molecular architecture of the amphiphiles by taking as an input a large set of molecular conformations, which are independently generated using a Monte Carlo method. The free-energy functional mentioned above depends on functions that describe the structure of the system and are unknown a priori, such as the local densities of the lipopeptides, ions and water molecules; the probability distribution function for lipopeptide conformations, and the position-dependent electrostatic potential and degree of protonation of each acid/base group in the lipopeptides. Minimization of the free energy functional with respect to these unknown functions and discretization in a lattice results in a set of coupled nonlinear equations that we solve using numerical methods.

The lipopeptide alkyl tails in MOLT are represented by tail beads (1 bead represents 4 CH_2_ units, so C_16_ is represented by 4 beads). Each amino acid is represented by a backbone bead and a side-chain bead. The parametrization of the model requires the bead volume and bead–bead interactions (see Tables S1 and S2 in the Supporting Information) to be defined. The latter are inspired by the MARTINI CG-MD force-field; i.e., each MOLT bead was assigned a MARTINI type and the interaction parameters were obtained from the MARTINI force field,^51,52^ as discussed in the Supporting Information of ref 33 (note that, unlike MARTINI, we use only one bead for all amino acid side chains). Two different parametrizations (“models” were tested), which differ in the short-range attractions, but had the same electrostatic interactions, acid–base properties (p*K*_a_) and steric repulsions (volumes); see Table S1. The differences in the short-range attractions result from the choice of the bead types used to represent different parts of the molecules. Briefly, in Model 1, backbone-K beads are type “P5” (polar and hydrophilic), while backbone-W and backbone-Y beads are “Nda”, which reflects the fact that W and Y have a higher propensity to form β-sheets than K.^53^ The W, Y and K side-chain beads are hydrophobic (“C5”, “C5” and “C3”, respectively, note that the interactions of the K-side-chain bead correspond to the neutral -NH_2_ state because electrostatic interactions are accounted for separately from short-range interactions). In Model 2, “Nda” is used for all backbone beads, and the K-side-chain bead is polar (“P1”) instead of hydrophobic. The parameters for tail, K-backbone and K-side chain beads used to model the behavior of C_16_-KK and C_16_-KKK as a function of pH in our previous works^33,36^ are those in Model 1, but both models reproduce correctly the experimental results for these lipopeptides as a function of pH.^33^

## Results and Discussion

Here we investigate the self-assembly of two pairs of model cationic lipopeptides, C_16_-KWK and C_16_-WKK and C_16_-KYK and C_16_-YKK (C_16_: hexadecyl or palmitoyl, W: tryptophan, K: lysine, Y: tyrosine). First the self-assembled nanostructure is determined by SAXS (complementing previously reported SAXS and cryo-TEM^38,54,55^ ), then the conformation is probed using FTIR spectroscopy. Having thus shown β-sheet ordering in fibril or bilayer nanotape structures depending on the sequence pattern, we then used a stastistical thermodynamics modeling approach (MOLT) that is able to predict the morphology and complemented this with atomistic MD to further elucidate details of the nanostructure, and molecular ordering and conformation.

SAXS data for the four lipopeptides are presented in Figure 2. It is immediately apparent that the data fall into two families. The intensity at low wavenumber *q* scales as *I* ∼ *q* ^–1^ for C_16_-YKK and C_16_-WKK and there is a sharp form factor maximum at high *q*. As described in our previous paper,^38^ these data can be well fitted using a core–shell cylinder model with a cylinder radius *R* = (16.5 ± 1.0) Å for C_16_-YKK and *R* = (13.6 ± 1.0) Å for C_16_-WKK (reasonable for a C_16_- lipid chain). The shell thickness is s = 10.0 Å or 14.6 Å for C_16_-YKK and C_16_-WKK respectively, and these latter values are reasonable for a tripeptide in an extended conformation. In marked contrast, the SAXS data at low *q* scale as *I* ∼ *q* ^–2^ for C_16_-KYK and C_16_-KWK (Figure 2) and the form factor at high *q* is broader and less intense than for the C_16_-YKK/C_16_-WKK cases. The data for C_16_-KYK and C_16_-KWK can be fitted to a form factor for a bilayer structure,^55^ consistent with the cryo-TEM observations which show twisted tape morphologies in solutions of these two lipopeptides.^55^ The bilayer thickness is (24.0 ± 2.0) Å for C_16_-KYK and (27.0 ± 4.0) Å for C_16_-KWK.

**Figure 2:**
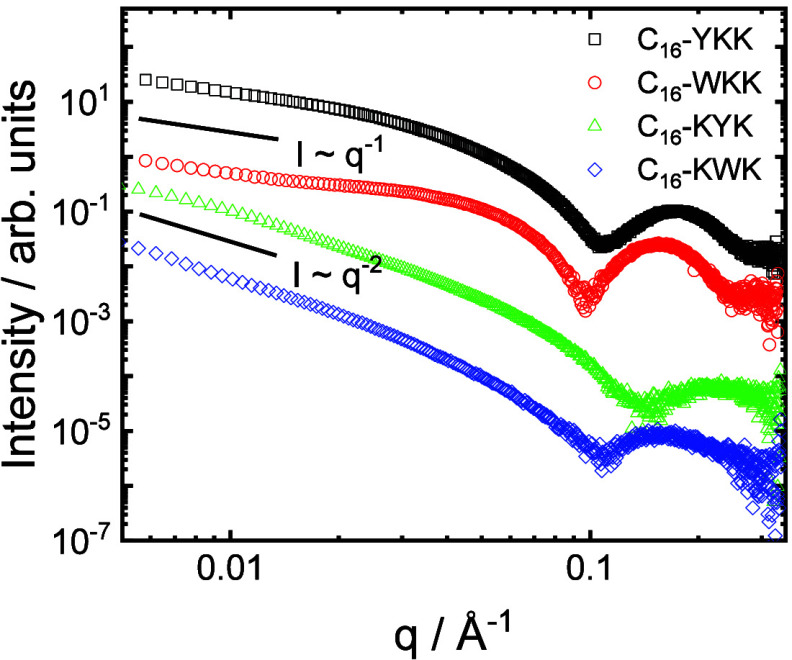
SAXS data for the lipopeptides (as indicated, 1 wt % solutions) at pH 8. Data sets have been shifted vertically (by multiplication by fixed factors) for ease of visualization.

FTIR spectroscopy was used to probe molecular conformation. The amide I′ region of the FTIR spectra shown in Figure 3 contains a peak for all samples at 1672 cm^–1^ due to bound TFA counterions.^56–58^ The peak in the range 1605–1622 cm^–1^ is a signature for β-sheet structure.^59,60^ The peak shifts significantly depending both on the nature of the aromatic residue (Y or W) and the sequence. It should be noted that Tyr has stretching and bending modes at 1603 cm^–1^ and 1612–18 cm^–1^ in D_2_O and Trp has stretching modes giving a peak at 1618 cm^–1^.^61,62^ This may distort the signal from the expected β-sheet structure for C_16_-WKK for which only a small peak is observed at this position, and may contribute to the observed shifts in peak position.^61,62^ Tyrosine also has a C–C stretching vibration peak at 1590–1591 cm^–1^ that gives rise to the shoulder peaks observed in the spectra for the Y-containing lipopeptides.^61,62^ The peak centered at 1455–1460 cm^–1^ has notable differences in absorbance (on an absolute basis and relative to the 1672 cm^–1^ TFA peak), being relatively stronger for C_16_-YKK and C_16_-WKK compared to the C_16_-KXK analogues. This peak is assigned to CH_2_/CH_3_ deformation modes in the lipid chain and/or lysine side chain.^60–62^ This peak may also contain contributions from tyrosine or tryptophan side chains in the 1450–1500 cm^–1^ range.^61,62^ The enhancement of this peak for C_16_-XKK samples may be associated with the fibril formation for these lipopeptides.

**Figure 3:**
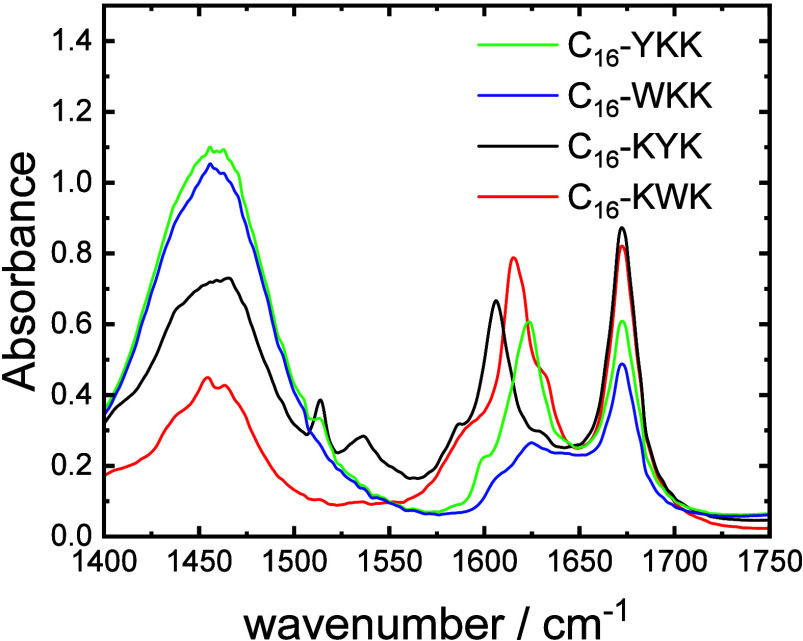
FTIR spectra for 1 wt % solutions as indicated.

MOLT has been shown to be a powerful tool for morphology prediction in model lipopeptides including C_16_-KK and C_16_-KKK.^33,36^ Here we compare the predictions of this theory with the experimental observations. Figure 4 shows the morphologies vs pH predicted by the two models detailed in the Methods section for C_16_-WKK and C_16_-KWK. The major difference between these models is that in Model 1 the backbones of W and Y form stronger H-bonds than K backbones, which accounts for the fact that W and Y have higher propensity to form β-sheets than K.^53^ In Model 2, all backbones are assumed to have identical interactions. In the high-pH limit, Model 1 correctly captures the experimental behavior (i.e. it predicts fibrils for C_16_-WKK^38^ and bilayers for C_16_-KWK),^55^ while Model 2 shows the opposite behavior. At low pH, both models predict micelles, which is consistent with experiments for C_16_-WKK,^38^ but not C_16_-KWK for which SAXS shows that nanotapes (with lamellar ordering) are stable over a range pH 2–12.^55^ Thus, Model 1 correctly captures the distinct morphologies formed by C_16_-WKK and C_16_-KWK at high pH. The breakdown in accuracy of Model 1 for C_16_-KWK at low pH is ascribed to an underestimation of the energy due to the cooperative nature of hydrogen bonding. This effect opposes the formation of micelles, which are favored by the strong electrostatic interactions that result from the protonation of the two lysines.

**Figure 4:**
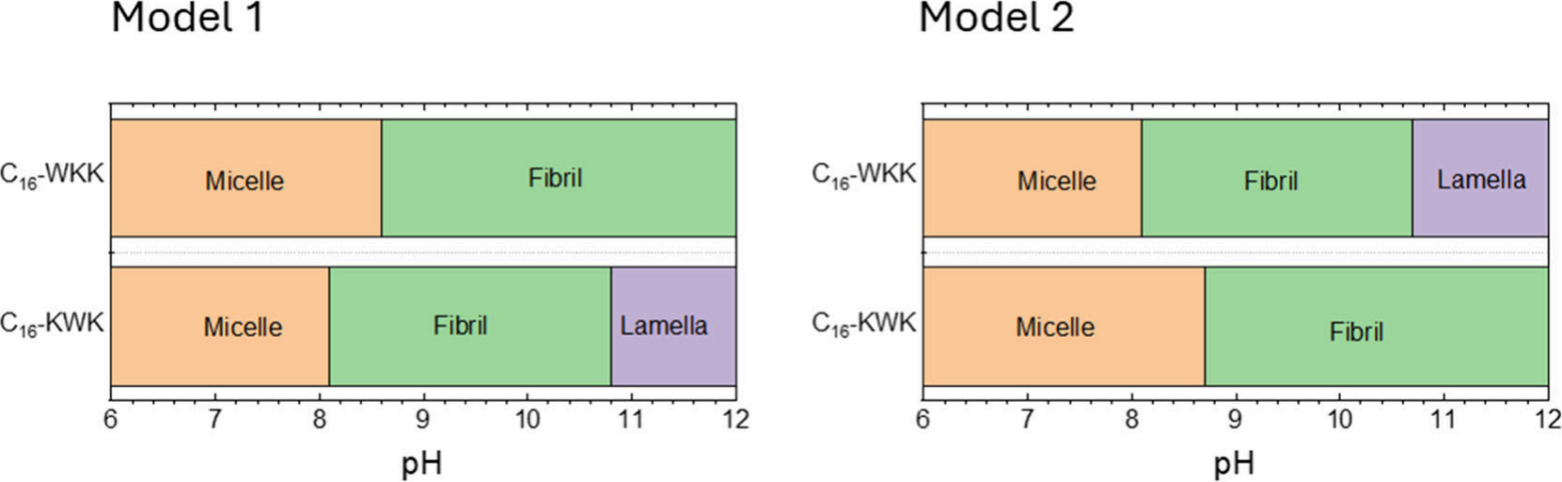
Morphology predictions for the two MOLT models for the two W-containing lipopeptides, with parameters in Table S1.

Figure 5 shows the volume fraction profiles for the bilayer structure for C_16_-KWK at pH 12 using Model 1, as well as fibers of C_16_-WKK under the same conditions. In these plots, the variable *r* is the distance to the central plane of the bilayer or to the central axis of the fibril, respectively. The peaks of the volume-fraction profiles in Figure 5 in the peptide region are consistent with the sequence, e.g., for C_16_-KWK the profiles show a maximum in the density of W between two maxima from K. In contrast the profiles for C_16_-WKK fibrils show peaks for W outside the lipid tail region, then there is an outer broad maximum for lysine residues. MOLT predicts that there is no water in the interior of the lipid region. Also notable is the extended lipid tail-rich region in the fibril interior for C_16_-WKK compared to that for the C_16_-KWK lamellae. This is a direct consequence of the geometry of the aggregate: in a cylindrical geometry (fibril), the volume element at distance *r* from the central axis is proportional to *r*; therefore, near the fibril axis (*r* = 0) there is less volume available than in the corona region. For a fixed number of lipopeptides, the radius of the core for the fibril is thus larger than the thickness of lamellae.

**Figure 5:**
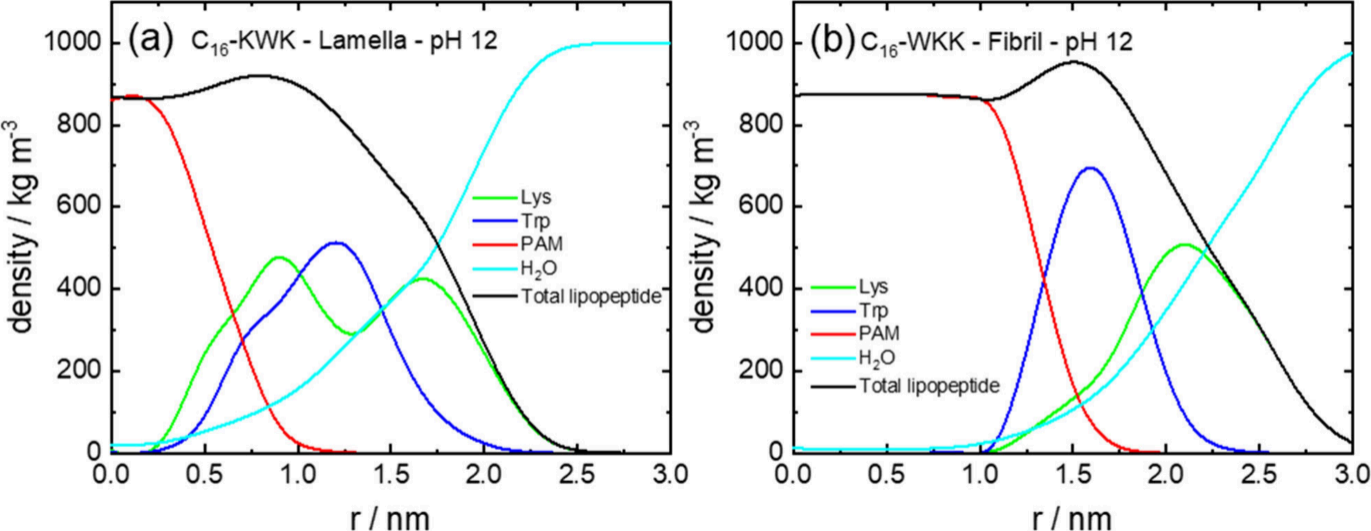
Density profiles from MOLT (Model 1) at pH 12 for (a) C_16_-KWK lamella and (b) C_16_-WKK fibril.

The parameters in Table S1 show that in our model, Y differs from W in two aspects: the volume of the Y side chain beads (0.153 nm^3^) is slightly smaller than that of W side chains (0.186 nm^3^) and, most important, the Y side chain has a phenol group that can be deprotonated, with p*K*_a_ 10.5. The morphology predictions using MOLT for C_16_-YKK and C_16_-KYK are presented in Figure 6. As for the W-containing analogue, the theory correctly predicts fibril formation at high pH for C_16_-YKK, but lamellae (pH 10.2–11.6) for C_16_-KYK (with a predicted re-entrant fibril morphology for pH 11.6–12.0). Also similar to the W-containing analogues, the model predicts micelles at low pH for both lipopeptides, although in experiments only C_16_-YKK shows this morphology.^38,55^ The model predicts fibrils for C_16_-KYK at pH > 11.6 because of the deprotonation of the phenol group in Y, which increases electrostatic repulsions, the stability of the bilayer morphology is then underestimated by the theory. Volume fraction profiles for C_16_-YKK and C_16_-KYK are presented in Figure 7 and show similar features to those for the W-containing analogues; i.e., the density maxima are in the expected sequence.

**Figure 6:**
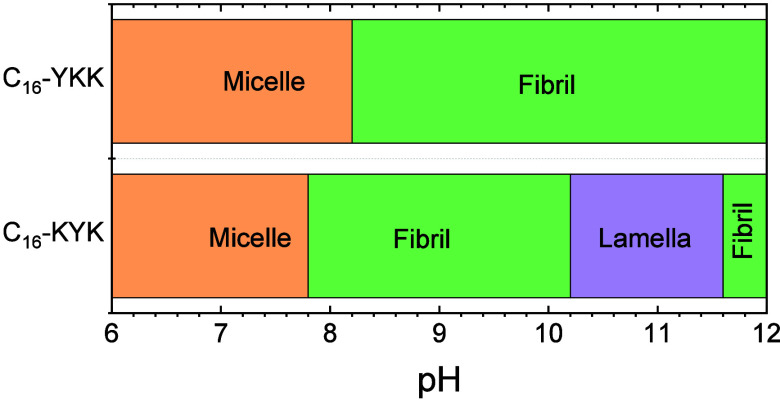
Morphology predictions for the two Y-containing lipopeptides using Model 1, with parameters in Table S1.

**Figure 7:**
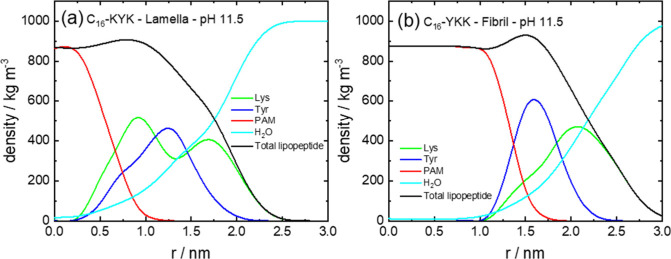
Volume fraction profiles from MOLT (Model 1) at pH 11.5 for (a) C_16_-KYK lamella, (b) C_16_-YKK fibril.

Fibril-to-micelle transitions in C_16_-WKK and C_16_-YKK upon decreasing pH are triggered by the protonation of the lysine side-chain, which results in strong electrostatic repulsions between the head groups. These repulsions favor the most curved morphology (micelles) over the least curved one (fibril). However, the predicted transition pH for C_16_-WKK (pH 8.6; see Figure 4) is lower than the p*K*_a_ of an isolated lysine (bulk p*K*_a_ = 10.54). The effect is mainly ascribed to charge regulation:^33^ the repulsions between protonated lysines shift the acid–base equilibrium toward the neutral state; therefore, the pH required to charge the fraction of lysines that is required to trigger the transition is lower than that expected from the bulk p*K*_a_. As a matter of fact, the apparent p*K*_a_ of lysine within C_16_-KKK aggregates measured by titration (p*K*_a_ 9.1) was found to be significantly lower than the p*K*_a_ of an isolated lysine in the bulk (p*K*_a_ 10.54).^33^ The fraction of protonated lysines required to trigger the transition is also an important parameter. For example, the transition pH predicted for C_16_-YKK (pH 8.2; see Figure 6) is lower than that for C_16_-WKK (pH 8.6, Figure 4) because the negative charge of tyrosine in C_16_-YKK increases the fraction of protonated lysines required for the transition (and thus lowers the transition pH).

The MOLT theory provides valuable predictions on lipopeptide morphology, which is very hard to access with atomistic MD, due to the computationally intensive nature of the simulations and the issue of systems becoming trapped in local free energy minima. However, atomistic MD can provide detailed information on conformation, orientation of the interactions and chirality that is not described by MOLT, and it is less susceptible to parametrization issues. Atomistic MD simulations were performed considering *a priori* the different modes of self-assembly (Figure 1). For C_16_-WKK and C_16_-YKK, the starting state was lipopeptide cylindrical fibrils with the peptides arranged in parallel β-sheets For C_16_-KWK and C_16_-KYK simulations the initial state comprises lipopeptide bilayers with the peptides arranged in parallel β-sheets. Extensive testing revealed that this method provides stable hydrogen bonded β-sheets. These were not obtained by simply randomly positioning molecules in layered structures, nor by constraining their orientation within such layers. The MD simulations were performed with 256 molecules which is sufficient to produce structures with extensive stable hydrogen-bonding along the fiber or nanotape axis. Images showing the configurations from the final frame of simulations are shown in Figure 8. The peptide backbones arranged perpendicular to the fibril/nanotape long axis are shown along with H-bonds, this being clearer in the enlarged images in Figure 8b,d,g,j. For C_16_-KWK and C_16_-KYK, since they form bilayer nanotapes, two projections of the structure are shown (Figure 8e–j).

**Figure 8:**
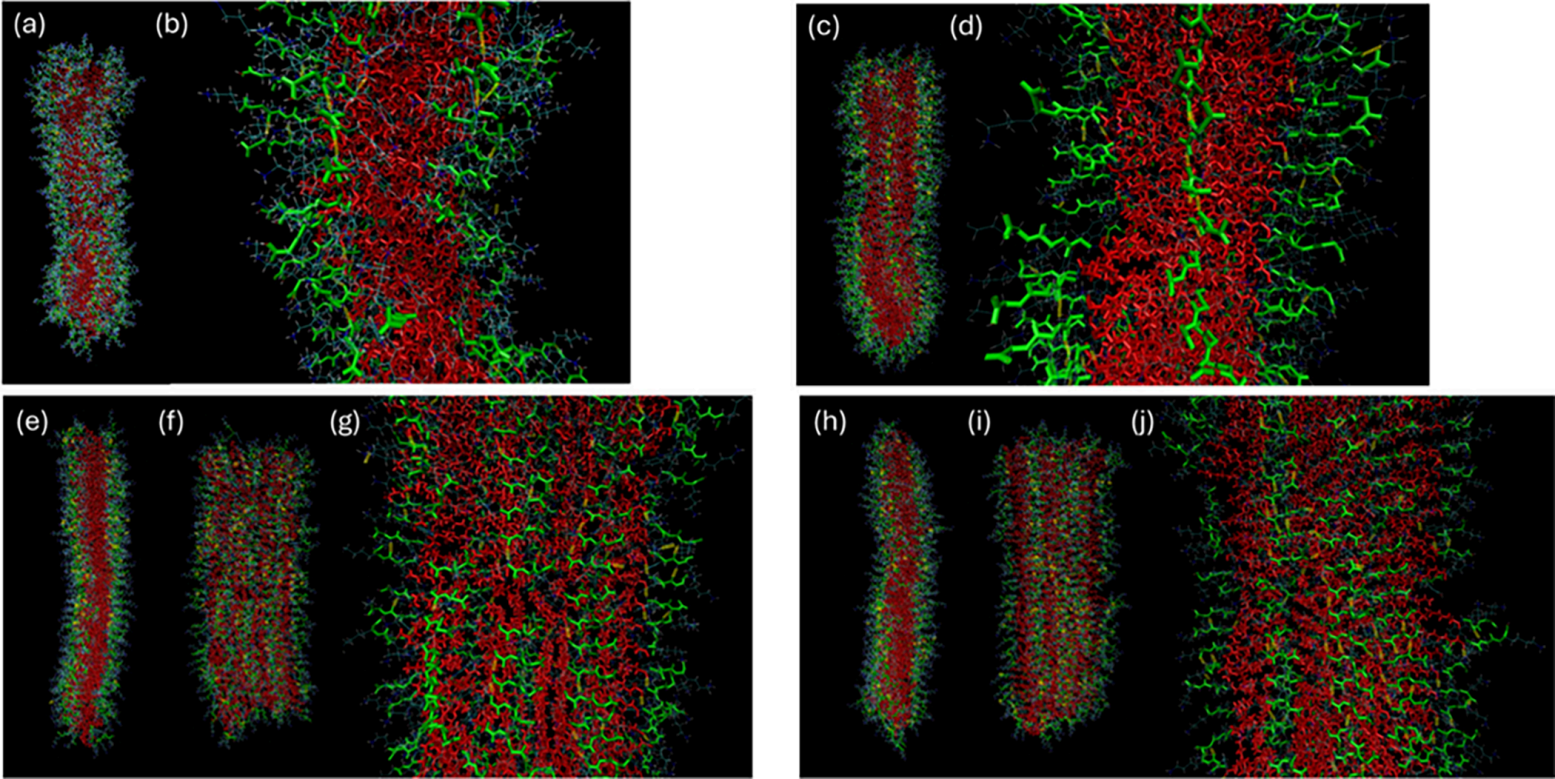
Configurations from MD simulation final frames. Thin blue/white lines: all bonds, Red: C_16_-chains. Green: peptide backbone. Yellow: H-bonds. (a, b) C_16_-WKK. The enlargement in part (b) shows backbones and H-bonds. (c,d) C_16_-YKK. The enlargement in part (d) shows backbones and H-bonds. (e,f,g) C_16_-KWK showing (e) side projection, (f) face projection. The enlargement in part (g) shows backbones and H-bonds. (h,i,j) C_16_-KYK showing (h) side projection, (i) face projection. The enlargement in part (j) shows backbones and H-bonds.

This modeling enables the production of bilayer structures with realistic density profiles across the layer. As shown by a representative density profile obtained for C_16_-KWK in Figure 9a, the bilayer is enriched in C_16_ (palmitoyl) units in the center, then there are broad density maxima corresponding to the K and W residues. These resemble qualitatively those calculated from the MOLT theory shown in Figure 5, although MOLT predicts a slightly greater segregation of W residues and yields a slightly larger lamellar thickness. The density profiles from MD indicate that the water is progressively excluded from the interior of the lipopeptide bilayer. However, it is interesting to mention that the density profiles for C_16_-KWK obtained from MOLT with Model 2 (i.e., the model that incorrectly predicts the high-pH behavior of the lipopeptides) has the W residues buried within the lipidic core (Figure S1 in the Supporting Information) and, therefore, those profiles strongly differ from those obtained by MD in Figure 9a. In comparison to the MOLT predictions in Figure 5 the atomistic MD indicates a significantly higher water content in the lipid interior. The simulated lipopeptide cylindrical fibril or bilayer structures are in quantitative agreement with measured SAXS profiles at high *q* (where the local structure in the fibril or bilayer is probed) as exemplified by data for C_16_-YKK and C_16_-KYK in Figure 9b. The SAXS profile was computed from the simulations by using a generated pdb file as input in CRYSOL, which was developed to calculate SAXS profiles from proteins, via the Debye equation using the atomic coordinates and allowing for the effects of displaced solvent and the hydration layer at the surface.^63–65^ We have recently shown that it can also be successfully used to calculate SAXS profiles for surfactant^66^ and lipopeptide^39^ micelles. The data in Figure 9b show that at low wavenumber *q* the simulations do not exhibit the same intensity scaling as in the measurements (Figure 2), which arises from to the extended fibrillar structure, either cylindrical fibrils for C_16_-YKK or bilayer nanotapes for C_16_-KYK. This is due to the finite width and length of the simulated ensembles.

**Figure 9:**
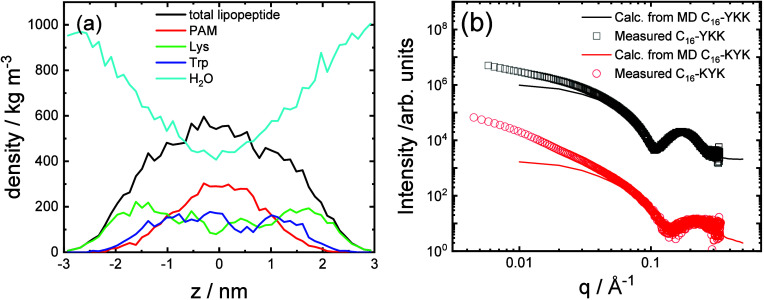
(a) Density profile across the lipopeptide bilayer calculated from final 100 ps of simulation for C_16_-KWK (PAM: C_16_) and comparison of simulated (last 100 ps of simulation) and experimental (1 wt % pH 8 solution) SAXS data for C_16_-YKK and C_16_-KYK. The data were scaled for ease of visualization and a constant background term was added to the calculated profiles.

The conformational free energy landscape of the lipopeptides was examined by Ramachandran plot analysis (Figure 10). Pairwise comparison shows that the β-sheet region is relatively favored for C_16_-WKK and C_16_-YKK, especially the former in comparison to the C_16_-KXK homologues. This suggests that β-sheet conformations are more highly favored in the cylindrical fibrils. The conformational landscape is similar for C_16_-KWK and C_16_-KYK, with significant populations sampling left and right-handed α-helical conformations as well as β-sheets. Further insight the structure of the assemblies is provided by analysis of hydrogen bonding. The data in Figure 11a show that while all lipopeptides show significant numbers of hydrogen bonds (more than 1.5 per molecule), there is a higher number for the two Y-containing lipopeptides, which reflects the fact that the tyrosine −OH is capable of forming H-bonds. On the other hand, the number of H-bond capable pairs within 0.35 nm is higher for the two C_16_-KXK lipopeptides than for the C_16_-XKK ones, reflecting the more favorable arrangements of pairs possible in bilayer nanotapes compared to cylindrical fibrils. The thermodynamic stability of the structures was also determined by evaluating the cohesive energy density^28^ which takes values CED = −1,144 kJ mol^–1^ nm^–3^ for C_16_-WKK, CED = −1,533 kJ mol^–1^ nm^–3^ for C_16_-YKK, CED = −1,530 kJ mol^–1^ nm^–3^ for C_16_-KWK, and CED = −1,520 kJ mol^–1^ nm^–3^ for C_16_-KYK. The values are similar, except the CED for C_16_-WKK is lower, probably reflecting the relatively lower stabilizing noncovalent interactions, i.e., hydrogen bonding and π–π stacking, observed for this lipopeptide.

**Figure 10:**
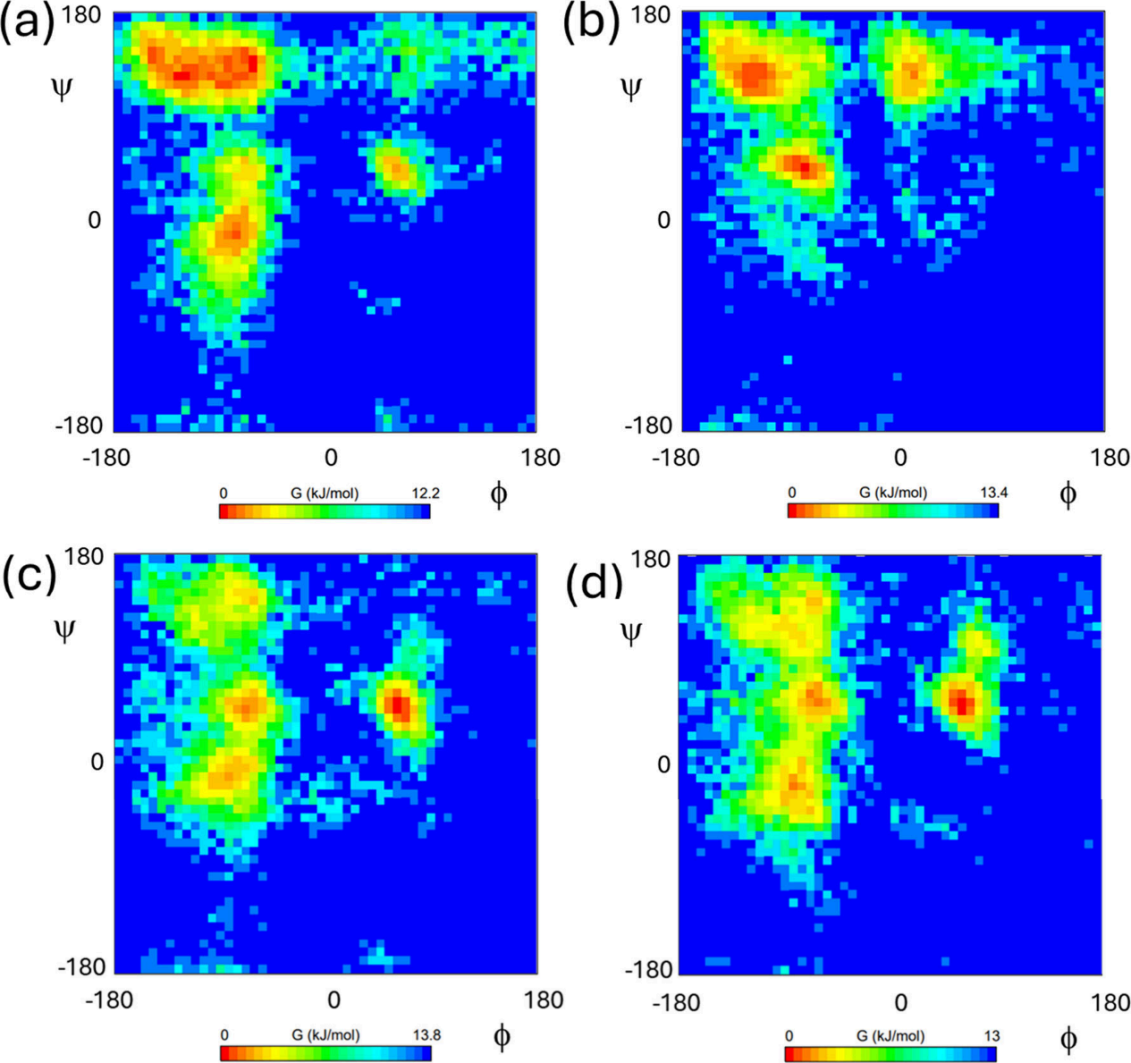
Ramachandran analysis (conformational free energy plots) from last 100 ps of MD simulations, (a) C_16_-WKK, (b) C_16_-YKK, (c) C_16_-KWK, (d) C_16_-KYK.

**Figure 11:**
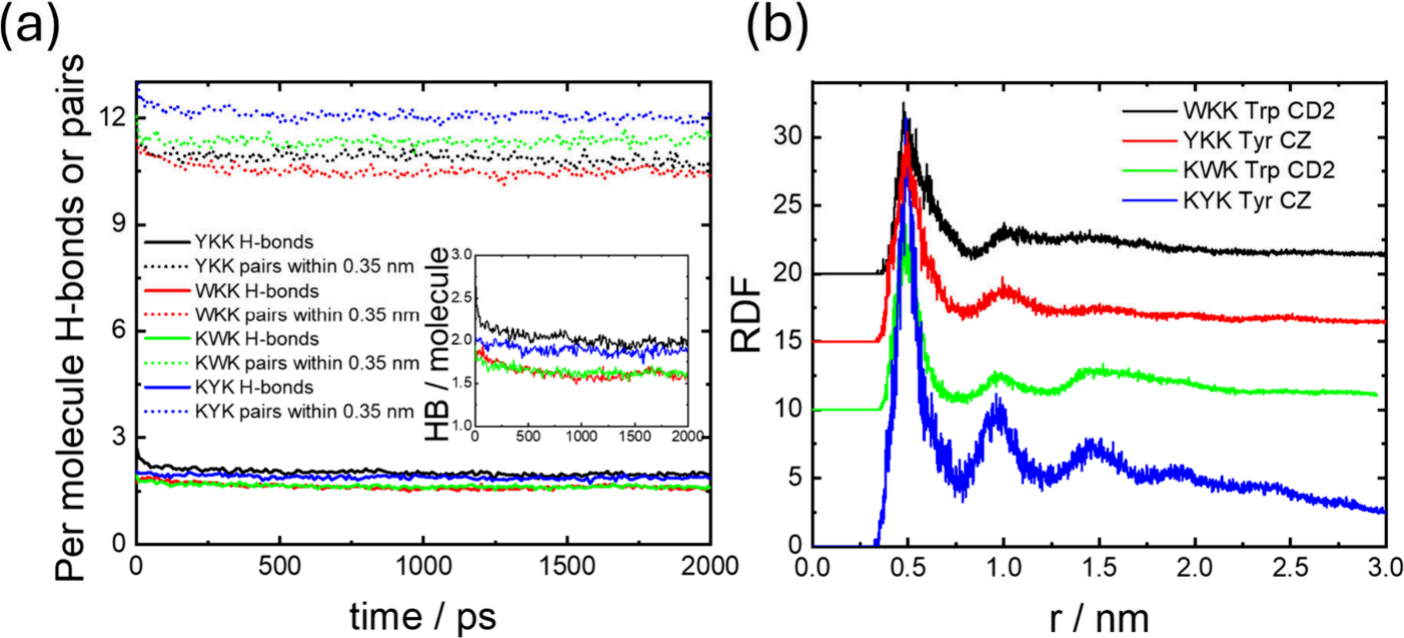
(a) Progression during simulation of per molecule numbers of hydrogen bonds and numbers of H-bond capable pairs of atoms within 0.35 nm for the lipopeptides as indicated. Inset: enlargement of H-bond number. (b) Radial distribution functions for aromatic groups (see Figure S2 for atom selections) from last 500 ps of simulations for the four lipopeptides. Data for C_16_-WKK, C_16_-YKK and C_16_-KWK is offset vertically for ease of visualization.

The self-assembly of the lipopeptides will also be substantially influenced by π–π stacking and this was examined via analysis of radial distribution functions for the aromatic groups in Trp and Tyr shown in Figure 11b (atom labeling scheme in Figure S2). This reveals more pronounced stacking of the aromatic residues in C_16_-KWK and C_16_-KYK which shows a periodic 5 Å stacking. The π-stacking is less extensive for the two fibril-forming lipopeptides C_16_-WKK and C_16_-YKK (Figure 11b).

The solvent-accessible surface area (SASA) and associated properties, i.e., free energy of solvation, Δ*G*(solv), and SASA-related volume and density were compared for the four lipopeptides. The data are shown in Figure S3 The aggregation propensity (AP) may be defined as the ratio of initial-to-final SASA,^26^ for C_16_-WKK AP = 1.53 and for C_16_-YKK AP = 1.49 which indicates high aggregation propensity for both of these fibril-forming lipopeptides. This may be contrasted with the behavior for C_16_-KWK and C_16_-KYK for which no trend for SASA to reduce is observed, instead the simulations show that after initial rapid equilibration, the SASA reaches a stable value (Figure S3).

## Conclusions

In summary, there are unexpected significant differences in the pH-dependent self-assembly of simple lipidated tripeptides depending on the sequence of the cationic lysine residues comparing C_16_-XKK and C_16_-KXK with X = W or Y. C_16_-WKK and C_16_-YKK form micelles at low pH (pH 3) but fibrils at high pH (pH 8) whereas C_16_-KWK and C_16_-KYK form lamellar nanotapes that are stable over a wide range of pH 2–12. Here we showed that MOLT is able to correctly predict the morphology at high pH, however at lower pH it incorrectly predicts micelle formation for the C_16_-KXK lipopeptides. This effect is ascribed to an underestimation of cooperative hydrogen bonding in C_16_-KXK by MOLT, which, therefore, fails to offset the electrostatic repulsions at low pH that favor micelle formation.^33^ The volume fractions obtained are physically realistic and suggest that the formation of C_16_-XKK lipopeptide fibrils may be promoted by incorporation of the X (=W or Y) residue into the fibril core. We also introduce a method to produce stable β-sheet assemblies for atomistic MD simulations, prepared a priori as lamellar nanotapes or fibrils. These simulations are able to probe differences in conformation and packing at an atomistic level, and importantly they reveal significantly higher aggregation propensity for the C_16_-XKK fibrils compared to C_16_-KXK lamellar nanotapes.

Based on the density profiles from MOLT, it is apparent that when W is close to the alkyl-chain region, fibers are favored over lamellae. On the contrary, when W or Y is located in the corona, lamellae are favored. The presence of W or Y in the corona increases the cohesiveness of the lipopeptide headgroup (due to the hydrophobic interactions between W or Y side chains and the strong hydrogen bonds between W or Y backbones). In terms of Israelachvili’s packing argument,^67,68^ the increase in cohesiveness of the headgroups decreases their effective size, favoring the least curved morphology (lamella).

There are notable differences in local interactions comparing the nanotape-forming C_16_-KXK lipopeptides with the fibril- forming C_16_-XKK analogues. The π-stacking is more pronounced in the former case. The radial arrangement of the backbones in the cylindrical fibrils in C_16_-WKK and C_16_-YKK reduces the extent of π–π interactions. On the other hand, the degree of hydrogen bonding depends primarily on the nature of the aromatic residue, being higher for the two lipopeptides bearing tyrosine compared to tryptophan, irrespective of nanostructure, due to the H-bonding capability of the Tyr hydroxyl group.

Future developments may include the use of generative AI to further predict β-sheet fibril formation of self-assembling peptides^69,70^ as well as to further understand the key molecular parameters that drive this, and that influence the detailed β-sheet structure.

## Supplementary Material



## Notes

Table and discussion of MOLT parameters, table of interaction parameters, density profiles from MOLT, figure of atom labeling, SASA from MD simulations (PDF)
