# Supplementary material for: Elucidating β‑Sheet Ordering in Lipopeptides Bearing Lysine-Rich Tripeptide Sequences: Fibrils versus Nanotapes
Source: J Phys Chem B. 2025 Dec 18;130(1):281–91. doi: 10.1021/acs.jpcb.5c06441 (PMC12794139; doi:10.1021/acs.jpcb.5c06441)
Supplement: Supplementary file 1 [file jp5c06441_si_001.pdf]

Supporting Information

Elucidating β-sheet Ordering in Lipopeptides Bearing Lysine-Rich Tripeptide Sequences: Fibrils versus Nanotapes

Ian W. Hamley,[1,*] and Valeria Castelletto,[1]

[1] *School of Chemistry, Food Biosciences and Pharmacy, University of Reading, Whiteknights, Reading RG6 6AD, U.K.*

Mario Tagliazucchi

INQUIMAE-CONICET and DQIAQF, University of Buenos Aires, School of Sciences, Ciudad Universitaria, Pabellón 2, Ciudad Autónoma de Buenos Aires C1428EHA, Argentina

Table S1. Parameters of the two MOLT models.

	Equivalent MARTINI bead for short-range attractions		Volume (nm^3) (same for both models)	Acid-base properties
	Model 1	Model 2		
Tail bead	C1	C1	0.113	
Backbone K	P5	Nda	0.113	
Backbone W	Nda	Nda	0.113	
Side chain K	C3	P1	0.128	Basic (pKa 10.54)
Side chain W	C5	C2	0.186	
Backbone Y	Nda	Nda	0.113	
Side chain Y	C5	C2	0.153	Acid (pKa 10.5)

Short-range interaction parameters in MOLT

Short-range attractions in MOLT are given by a term of the form:[1]

$$\beta F_{short-range} = -\frac{1}{2}\sum_{\substack{\text{i=all bead}\\ \text{types}}} \sum_{\substack{\text{j=all bead}\\ \text{types}}} \int\int \langle n_i(\boldsymbol{r})\rangle \langle n_j(\boldsymbol{r}')\rangle \beta\varepsilon_{ij} g(|\boldsymbol{r}-\boldsymbol{r}'|) d\boldsymbol{r} d\boldsymbol{r}',$$

where $\langle n_i(\mathbf{r})\rangle$ is the density of beads of type i at position $\mathbf{r}$, $\beta = 1/k_B T$, and $g(d)$ is a function that accounts for the dependence of the interaction with distance,

$$g(d) = \begin{cases} \left(\frac{a}{d}\right)^6 & a < d < d_{cutoff} \\ 0 & otherwise \end{cases},$$

(where we used a = 0.5 nm and d_{cutoff} = 1.0 nm). Finally, ε_{ij} is the effective strength of the interaction between a bead of type i and a bead of type j. These parameters are obtained by assigning MARTINI types to each bead in the system and then using the procedure described in the SI of Ref. 1 to obtain the parameters ε_{ij} required by MOLT from the Lennard-Jones parameters used by MARTINI. Note that while in MARTINI there are water beads, the solvent in MOLT is implicit for the purpose of short-range interactions, therefore the parameters ε_{ij} are effective interaction parameters (i.e., they represent the strength of the interaction between two beads in water). Table S2 shows the ε_{ij} matrices used for all calculations in the paper. Note that because ε_{ij} are effective interaction parameters, there are negative values in the table, which corresponds to effective repulsions between beads.

Table S2. Interaction parameters $\beta\varepsilon_{ij}$ used in the calculations.

Model 1 – W lipopeptides					
	Tail bead	Backbone K	Backbone W	Side chain K	Side chain W
Tail bead	7.00	-0.93	2.64	6.70	7.68
Backbone K	-0.93	-0.93	0.62	-1.06	-1.54
Backbone W	2.64	0.62	2.33	1.76	3.58
Side chain K	6.70	-1.06	1.76	6.19	7.83
Side chain W	7.68	-1.54	3.58	7.83	9.69
Model 2 – W lipopeptides					
	Tail bead	Backbone K	Backbone W	Side chain K	Side chain W
Tail bead	7.00	2.64	2.64	2.11	7.40
Backbone K	2.64	2.33	2.33	1.76	2.47
Backbone W	2.64	2.33	2.33	1.76	2.47
Side chain K	2.11	1.76	1.76	1.00	2.59
Side chain W	7.40	2.47	2.47	2.59	7.78
Model 1 – Y lipopeptides					
	Tail bead	Backbone K	Backbone Y	Side chain K	Side chain Y
Tail bead	7.00	-0.93	2.64	6.70	6.32
Backbone K	-0.93	-0.93	0.62	-1.06	-1.26
Backbone Y	2.64	0.62	2.33	1.76	2.95
Side chain K	6.70	-1.06	1.76	6.19	6.44
Side chain Y	6.32	-1.26	2.95	6.44	6.56

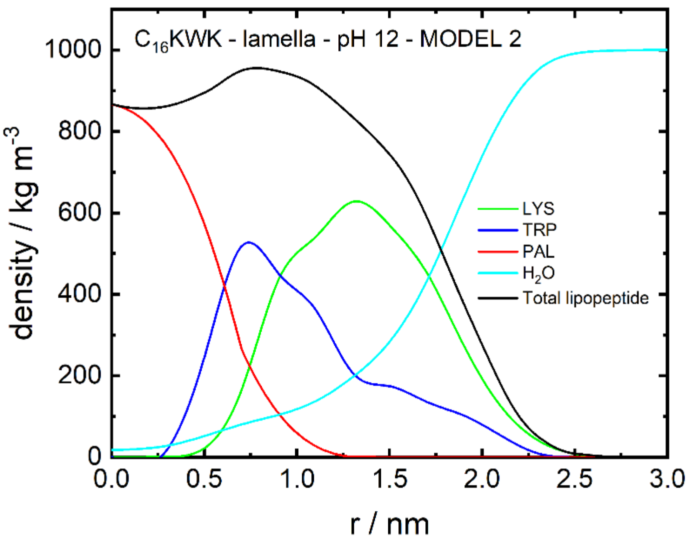

Figure S1. Density profiles from MOLT (Model 2) at pH 12 for C_{16}-KWK lamella. Note that W is partly buried in the C_{16} core because of the choice of interaction parameters in Model 2, which incorrectly captures the experimental morphology of the system (Fig.4 in the main text).

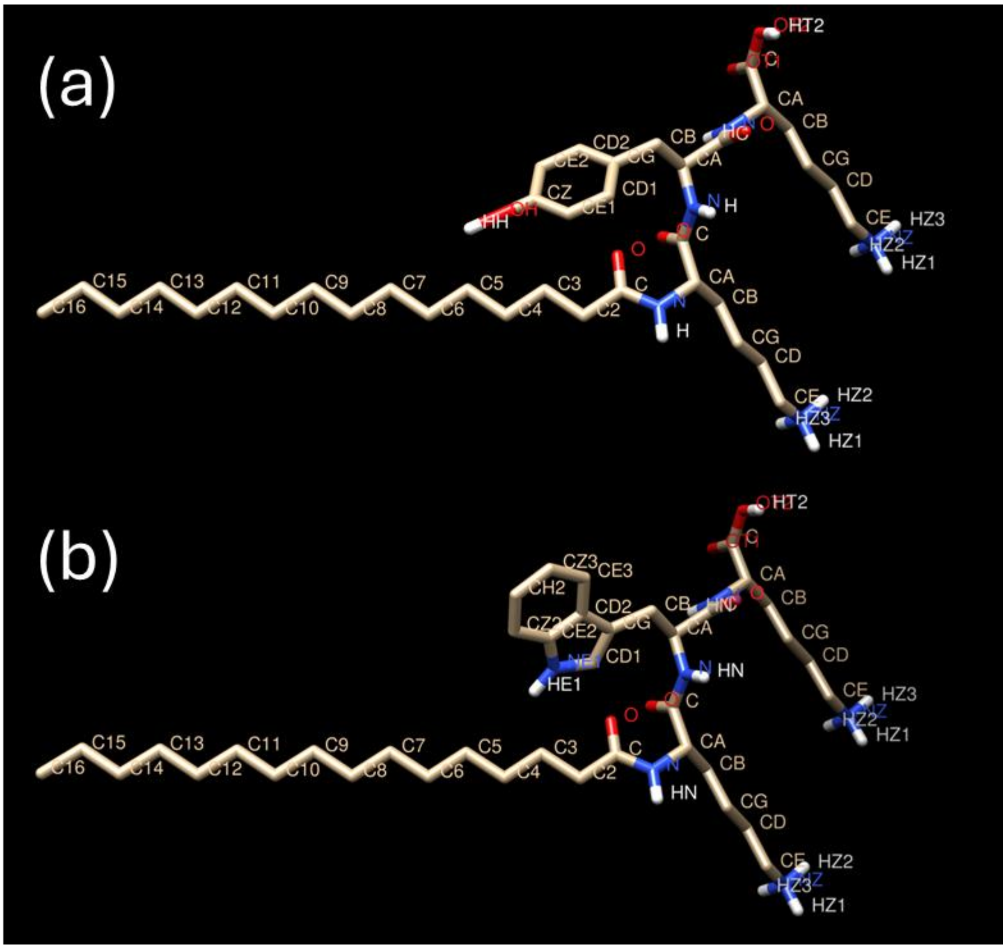

Figure S2. Atom labelling for aromatic group RDF calculations. (a) Atom labelling for W-containing lipopeptides, RDF computed using CD2 (b) Atom labelling for Y-containing lipopeptides, RDFs computed using CZ. The same labelling was used in ref.[2] Backbone hydrogen atoms are not shown for convenience.

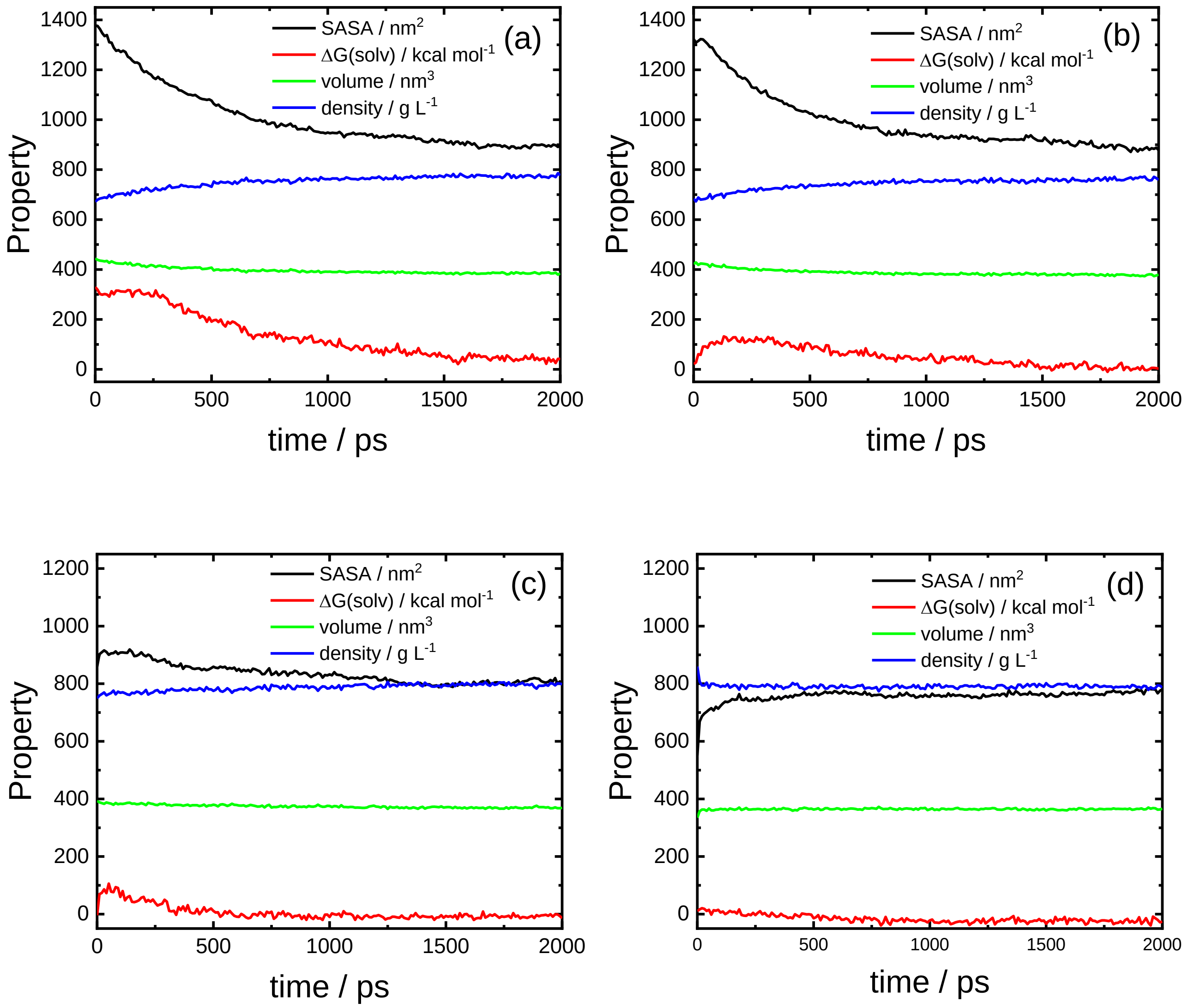

Figure S3. SASA and related quantities ΔG(solv), volume, density. (a) C_{16}-WKK, (b) C_{16}-YKK, (c) C_{16}-KWK, (d) C_{16}-KYK.

References

(1) Zaldivar, G.; Vernulapalli, S.; Udumula, V.; Conda-Sheridan, M.; Tagliazucchi, M., Self-Assembled Nanostructures of Peptide Amphiphiles: Charge Regulation by Size Regulation. *Journal of Physical Chemistry C* **2019,** *123* (28), 17606-17615.

(2) Hamley, I. W.; Adak, A.; Castelletto, V., Lysine-Rich Lipopeptide Micelles: Influence of Chirality and Sequence in Model Colloidal Systems and Biosurfactants. *Nature Commun.* **2024,** *15,* 6785.
